# Superior mesenteric vein thrombosis as uncommon complication following laparoscopic sleeve gastrectomy: a case report and literature review

**DOI:** 10.1093/jscr/rjaf441

**Published:** 2025-06-25

**Authors:** Mones H Atatre, Mahdi W Suboh, Ala'a S Ghnimat, Bessan Hamed Dababseh, Orwa Z Al-Fallah, Izzeddin Ghazi Eqtait, Ahmad G Hammouri, Ibraheem AbuAlrub

**Affiliations:** Department of Clinical Medical Sciences, Faculty of Medicine and Health Sciences, Palestine Polytechnic University, Hebron, Palestine; Department of Clinical Medical Sciences, Faculty of Medicine and Health Sciences, Palestine Polytechnic University, Hebron, Palestine; Department of Clinical Medical Sciences, Faculty of Medicine and Health Sciences, Palestine Polytechnic University, Hebron, Palestine; Department of Clinical Medical Sciences, Faculty of Medicine and Health Sciences, Palestine Polytechnic University, Hebron, Palestine; Radiology Department, Al-Ahli Hospital, Hebron, Palestine; Hematology Department, Al-Ahli Hospital, Hebron, Palestine; Radiology Department, Al-Ahli Hospital, Hebron, Palestine; Department of Clinical Medical Sciences, Faculty of Medicine, Palestine Polytechnic University, Hebron, Palestine

**Keywords:** laparoscopic sleeve gastrectomy, superior mesenteric vein thrombosis, anticoagulation therapy, morbid obesity, portal thrombosis

## Abstract

Laparoscopic sleeve gastrectomy (LSG) is a common bariatric procedure with relatively low complication rates. However, portomesenteric venous thrombosis (PMVT) is an uncommon serious complication that can occur after surgery. If not diagnosed early, this condition can lead to intestinal ischemia. We present the case of a 49-year-old woman with obesity (BMI 60.9 kg/m^2^), hypertension, and diabetes mellitus, who developed PMVT 20 days after undergoing LSG. She presented with severe epigastric pain, nausea, and diarrhea. Imaging revealed thrombosis in the superior mesenteric and portal veins, laboratory tests indicated multiple prothrombotic factors, including factor V Leiden mutation and reduced Antithrombin III levels. The patient was managed with anticoagulation therapy and was discharged on warfarin with close INR monitoring. This case highlights the critical importance of early detection and effective treatment of PMVT after LSG. A multidisciplinary approach, including hematology and vascular surgery consultations, is crucial to prevent complications and improve patient outcomes.

## Introduction

Laparoscopic sleeve gastrectomy (LSG) has emerged as one of the most popular bariatric surgeries due to its proven effectiveness in weight loss and its relatively low complication rates [1, 2]. Large portions of the stomach are removed during this treatment, which reduces stomach capacity and encourages weight reduction and early satiety [2]. Portomesenteric venous thrombosis (PMVT) is one of the uncommon but serious complications linked to LSG, despite its advantages. A thrombus in the portal or mesenteric veins causes PMVT, which can result in potentially fatal diseases, such as intestinal infarction or mesenteric ischemia [1, 3]. Although the prevalence of PMVT after LSG is thought to be between 0.3% and 0.4%, the consequences can be rather serious, with mortality rates ranging from 20% to 50% [2, 3].

Hypercoagulable conditions, increased pressure in the abdominal cavity during the procedure, dehydration, and venous stasis in the splanchnic circulation, are believed to be some of the factors that contribute to the development of PMVT [1, 2]. Nonspecific symptoms, such as nausea, vomiting, gastrointestinal discomfort, and vague abdominal pain, are common when patients presented [3]. Since these symptoms frequently coexist with other prevalent postoperative disorders, early identification may be difficult. Computed tomography (CT) scan is the most diagnostic method; it has a sensitivity rate of around 90% for venous thrombosis detection [1, 3]. The main treatment for PMVT is anticoagulant therapy, which resolves the problem without operations [1]. Nonetheless, bowel resection and other surgical procedures may be required in situations where complications, such as intestinal ischemia arise [1, 3].

## Case

A 49-year-old morbidly obese female (BMI 60.6 kg/m^2^), diabetic, hypertensive, and ex-smoker presented to the emergency department 4 days after onset of abdominal pain. The pain was described as colicky epigastric pain that began suddenly and radiated to the back, elevated by analgesia. The pain was accompanied by nausea and diarrhea, but denied fever, chest pain, chills, shortness of breath, vomiting, or dysuria. The patient underwent LSG 20 days prior to onset of symptoms.

Upon arrival, her vital signs were within normal limits: heart rate was 97 beats per minute, and blood pressure of 123/88 mmHg, and all other vital signs were stable. On physical examination, her abdomen was soft with no tenderness, but there was noticeable abdominal distension.

Laboratory tests revealed an elevated white blood cell count of 11 400/μl and significant raised plasma D-dimer level of 3500 ng/ml. The international normalized ratio (INR) was 1.1. Hemoglobin was 14.5 g/dl, and platelets count was 427 000/μl. Other laboratory tests were unremarkable.

An abdominal ultrasound revealed a non-distended gallbladder and free of stones, however a semi-solid sludge was noted. The patient was initially managed with triple therapy (proton pump inhibitor, Pramin, and Scobutyl). The patient’s symptoms improved, and she was subsequently discharged from the hospital. However, 4 days after discharge, she returned to ED with similar severe abdominal pain and was admitted to the hospital for additional assessment and care.

**Figure 1:**
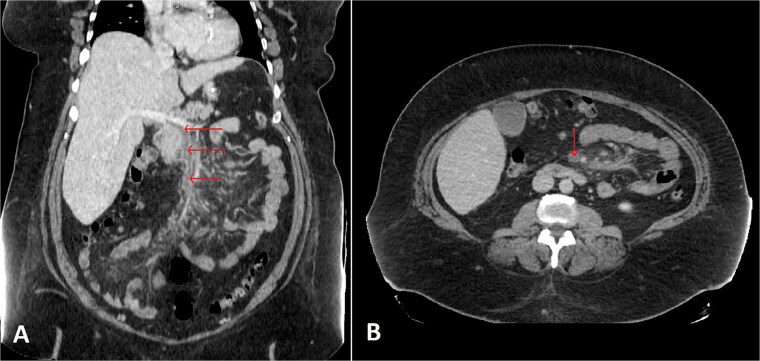
Selected coronal (A) and axial (B) cuts of the patient’s abdomen CT scan with IV contrast showing intra-luminal filling defect in the SMV, extending proximally to the proximal part of the main portal vein (arrows) associated with surrounding mesenteric fat haziness, representing portomesenteric venous thrombosis.

A contrast-enhanced abdominal and chest CT scan (Fig. 1) revealed an intra-luminal filling defect in the superior mesenteric vein (SMV), extending proximally to the main portal vein, associated with mesenteric fat haziness along the SMV and its tributaries. These findings were consistent with portomesenteric venous thrombosis (PMVT). The patient was admitted to the medical ward, kept NPO (nothing by mouth), and further blood tests were ordered. The results revealed the patient to be heterozygous for factor V Leiden, with low levels of antithrombin III, a beta-fibrinogen mutation (455G>A), and the MTHFR C677T gene mutation.

The patient was initiated on the maximum dose of Clexane (enoxaparin) twice daily alongside analgesic therapy. Consultations were obtained from hematology and vascular surgery team. A lower extremity venous duplex ultrasound was conducted, which ruled out deep venous thrombosis (DVT). A follow-up CT scan with contrast showed no significant changes regarding the presence of SMV and portal thrombosis.

After a 10-day hospital stay, the patient was discharged on a regimen of warfarin, starting with 5 mg once daily on days 1 and 2, followed by 7.5 mg on Day 3. She was advised to attend follow-up appointment at the outpatient clinic for close monitoring of her INR with a target range of 2–3 every 3 days. The continuation of anticoagulation therapy was planned based on further clinical evaluation and findings.

## Discussion

LSG has gained widespread popularity as a primary bariatric treatment that is becoming more popular by both patients and surgeons. During this treatment, more than 75% of the stomach’s greater curvature is removed, resulting in a tubular structure with a residual volume of ~100 ml [2]. Thrombosis of the portal vein is an uncommon yet potentially fatal complication of LSG [4].

Many risk factor that found to be associated with the elevated risk of PMVT following LSG, which includes venous flow stasis, due to increased intra-abdominal pressure during abdominal insufflation by CO_2_, manipulation of splanchnic vessels, and the hypercoagulable state often associated with obesity. These factors are assumed to contribute to formation of a thrombus in the mesenteric venous system [5]. Obesity stimulates chronic inflammation, which may increase the levels of coagulation factors in plasma, such as fibrinogen, von Willebrand factor, and factor VIII, further increasing the risk for thrombotic events. The inflammatory cytokines released by adipose tissue influence hepatocytes and endothelial cells, thus potentially exacerbating thrombosis risk postoperatively [6].

In this case, the patient exhibited several of these risk factors, including obesity (BMI 60.9 kg/m^2^), hypertension, diabetes mellitus, and the surgical manipulation of the splanchnic vessels during LSG. Other contributing factors could also have included prolonged surgical time and the venous stasis from the procedure. Interestingly, PMVT is usually asymptomatic in the initial stages, which may explain why most patients have a variety of non-specific symptoms, such as abdominal pain, nausea, vomiting, and even indications of intestinal infarction [7]. For this case, the patient had symptoms of bloating and absence of bowel movements, which are common in such cases and warrant early suspicion for this complication. The definitive diagnosis is usually through contrast-enhanced CT scans and Doppler ultrasound studies that confirm the presence and extent of thrombosis in the mesenteric veins [4].

Management of PMVT often involves anticoagulation for the prevention of further thrombotic events and the resolution of the thrombus. The treatment usually contains LMWH, such as enoxaparin (Clexane) [8]. For obese patients, the standard dose of enoxaparin treatment for venous thromboembolism is 1 mg/kg based on actual body weight is recommended [9].

Guidelines recommend a duration of 6–12 weeks of full-dose therapeutic anticoagulation for most patients with PVT, tailoring the treatment approach to be in line with the management of proximal deep vein thrombosis, In high-risk individuals or those with other thrombotic risk factors (e.g. previous history of DVT), prolonged anticoagulation therapy may be beneficial [10].

In this case, the patient after her hospital stay, was switched to warfarin, maintaining close control of her INR levels as guidelines recommend that obese patients on anticoagulants should be managed with vitamin K antagonists (VKAs), such as warfarin, which is often preferred over direct oral anticoagulants (DOACs) for long-term anticoagulation, especially in bariatric surgery patients. This is due to the limited evidence regarding the safety and pharmacokinetics of DOACs in this population. VKAs, on the other hand, are widely used because of the ability to monitor therapeutic levels through the INR, making them more predictable and safer choice until more research is available [11].

## Patient perspective

The patient was concerned regarding her overall health and expressed her fear of death. She was reassured about her general condition and referred to the psychology team in the hospital.
